# Global Myocardial Strain in Multisystem Inflammatory Syndrome in Children, Kawasaki Disease, and Healthy Children: A Network Meta-Analysis

**DOI:** 10.3389/fped.2022.848306

**Published:** 2022-06-27

**Authors:** Kaiwei Liu, Jiahui Yu, Guang Song

**Affiliations:** Department of Ultrasound, Shengjing Hospital of China Medical University, Shenyang, China

**Keywords:** systemic inflammatory response syndrome, pediatric multisystem inflammatory disease, COVID-19 related, mucocutaneous lymph node syndrome, myocardial strain, meta-analysis

## Abstract

**Background:**

Nearly 6,000 multisystem inflammatory syndrome in children (MIS-C) have been reported in the United States by November 2021. Left ventricular global myocardial strain has been proved to be one of the best evidence of the diagnostic and prognostic implications for cardiac dysfunction. The global myocardial strain change of MIS-C in the acute phase was still unclear.

**Methods:**

PubMed and other sources were searched. A network meta-analysis was conducted. MIS-C was divided into two groups according to left ventricular ejection fraction (LVEF): MIS-C with depressed ejection fraction (MIS-C dEF) and MIS-C with preserved ejection fraction (MIS-C pEF). Global longitudinal strain (GLS) and global circumferential strain (GCS) were compared among MIS-C, Kawasaki disease (KD), and healthy children.

**Results:**

In total, nine case-control studies were included, published between 2014 and 2021. These studies involved 107 patients with MIS-C, 188 patients with KD, and 356 healthy children. After Bayesian analysis, MIS-C dEF group was found to have a lower LVEF, higher GLS and GCS than the KD groups. Both MIS-C pEF and KD had similar GLS and GCS, which were higher than healthy controls. There was no difference of LVEF among MIS-C pEF, KD, and healthy controls.

**Conclusion:**

MIS-C dEF was more severe than KD, both in LVEF and global myocardial strain. MIS-C pEF and KD were similar with mild impaired left ventricular myocardial strain compared with the healthy children. Global myocardial strain may be a monitoring index for MIS-C.

**Systematic Review Registration:**

[https://www.crd.york.ac.uk/prospero/], identifier [CRD42021264760].

## Introduction

As of December 21, 2021, 273 million confirmed Coronavirus Disease-19 (COVID-19) cases, and 5.3 million deaths were reported globally (1). Centers for Disease Control and Prevention (CDC) issued an advisory in May 2020 about the symptoms of the multisystem inflammatory syndrome in children (MIS-C) which is characterized by a hyperinflammatory syndrome with multiorgan dysfunction (2). This syndrome is also known as pediatric inflammatory multisystem syndrome temporally associated with SARS-CoV-2, or pediatric multisystem inflammatory syndrome.

The current estimated incidence of MIS-C was estimated to be approximately 316 per 1,000,000 individuals < 21 years of age infected with COVID-19 (3). By the end of November 2021, CDC has received reports of nearly 6,000 MIS-C cases in the United States (4). More than 80% of patients with MIS-C present with cardiac involvement, including cardiac dysfunction, myocarditis, coronary artery abnormalities, valve regurgitation, pleural effusions, and cardiac arrhythmias (5).

Recently published articles summarize various similarities between MIS-C and Kawasaki disease (KD) (6, 7). Among children in developed countries, KD is the most common form of acquired cardiac disease (8). The estimated incidence of 250 cases per 1,000,000 children < 5 years in the North America. KD can lead to infiltration of inflammatory cells into medium-sized arteries, particularly the coronary arteries, with a risk of myocardial infarction and sudden death. In the acute phase, both MIS-C and KD have elevated cardiac markers, such as N-terminal pro-brain natriuretic peptide and troponin (9). Both MIS-C and KD could affect myocardial function in the acute phase (10, 11).

To assess the myocardial injury of MIS-C, speckle-tracking echocardiography (STE) with myocardial strain would be an ideal tool. Myocardial strain is a useful index of left ventricular deformation. The left ventricular global strain provides one of the best evidence on the diagnostic and prognostic implications for cardiac dysfunction (12). Some studies compared the myocardial strain using STE between MIS-C and KD (13, 14). Others compared the myocardial strain using STE between MIS-C and healthy children (15, 16). However, there was some inconsistency between the results, and sample sizes can affect the quality of evidence. In the scarcity of directly comparing studies, a network meta-analysis allows head-to-head comparisons indirectly through a common comparison group. Therefore, we conducted this network analysis to explore the myocardial strain of MIS-C.

## Methods

The pre-registered protocol was implemented in the PROSPERO database (CRD42021264760). This manuscript was reported in accordance with PRISMA guideline (Supplementary Material).

### Search Strategy

PubMed, MEDLINE, Web of Science, Embase, China national knowledge infrastructure (CNKI), and medRxiv.org from database were searched on 1 December 2021, to identify the relevant studies by two investigators. Keywords in the search strategy were (“Multisystem Inflammatory Syndrome” or “Kawasaki disease”), “pediatrics,” and “global myocardial strain” (Supplementary Table 1). In addition, we read the references of articles in search for literature that meets the criteria.

### Data Extraction and Extraction

Original studies were eligible if the following criteria were met: (i) observational study in English or Chinese; and (ii) compared the global left ventricular myocardial strain [including global longitudinal strain (GLS), global circumferential strain (GCS)] among patients with MIS-C and KD and healthy children.

Original studies were ineligible if the following criteria were met: (i) global myocardial strain obtain using other methods, not speckle-tracking echocardiography; (ii) data acquired from KD patients were not during the acute phase before treatment; or (iii) single-arm study of MIS-C or KD.

The first author, the published year, number of participants in each group, median, global left ventricular myocardial strain, and left ventricular ejection fraction (LVEF) were extracted in the involved eligible studies.

### Statistical Analysis

Before analysis, the quality of involved studies was evaluated using the Newcastle–Ottawa Scale. A score with a range of 0–9 was allocated to each study. Mean differences (MDs) and 95% CI were used to report the global myocardial strain and LVEF. We evaluated the myocardial strain using network meta-analysis. According to whether LVEF was normal or not, previous studies divided MIS-C into two groups: MIS-C with depressed ejection fraction (MIS-C dEF) and MIS-C with preserved ejection fraction (MIS-C pEF) (17–19). In this Bayesian network meta-analysis, a random-effects model and consistency model were applied (4 chains, 50,000 iterations, and 20,000 per chain). Inconsistency was assessed by the node-splitting method with Bayesian *P*-value. We analyzed the symmetry of a comparison-adjusted funnel plot to evaluate possible small sample effects, and used the Begg’s and Egger’s tests to evaluate publication bias in the included studies. A *p*-value < 0.05 was considered statistically significant for asymmetry. All the analyses were performed with the “gemtc” package in R software (R Foundation, Vienna, Austria, 4.0.2) and Stata (version 16.0; StataCorp, College Station, TX, United States).

## Results

**FIGURE 1:**
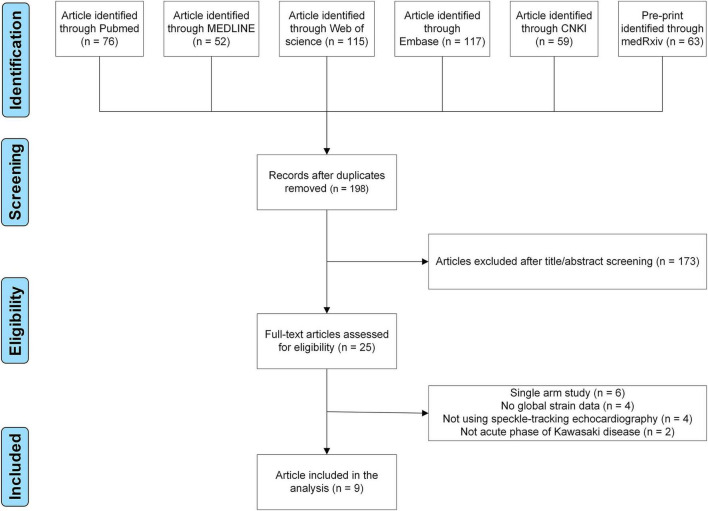
Flow chart of study selection.

**FIGURE 2:**
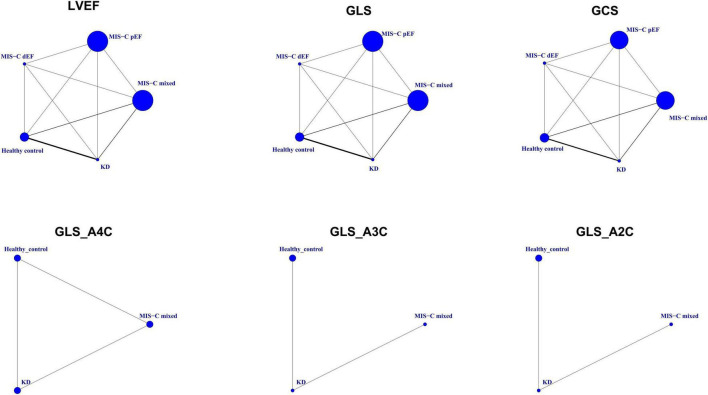
Geometry of the network. Circles represent the intervention as a node in the network, size of circles corresponds to the number of participants included in each comparison, lines represent direct comparisons using studies, and the thickness of lines corresponds to the number of studies included in each comparison. GCS, global circumferential strain; GLS, global longitudinal strain; GLS_A2C, global longitudinal strain_apical two-chamber; GLS_A3C, global longitudinal strain_apical three-chamber; GLS_A4C, global longitudinal strain_apical four-chamber; KD, Kawasaki disease; LVEF, left ventricular ejection fraction; MIS-C, multisystem inflammatory syndrome in children; MIS-C dEF, Multisystem Inflammatory Syndrome in Children with depressed ejection fraction; MIS-C pEF, Multisystem Inflammatory Syndrome in Children with preserved ejection fraction.

Finally, nine case-control studies were included (Figure 1; 13–16, 20–24). Evaluation of the quality of involved studies is presented in Supplementary Table 2. These nine studies conducted between 2014 and 2021 involved 651 participants: 107 patients with MIS-C, 188 patients with KD, and 356 healthy children (Table 1). Geometry of the network is shown in Figure 2.

**TABLE 1:** Characteristics of involved studies and comparisons of global myocardial strain among multisystem inflammatory syndrome in children, Kawasaki disease, and healthy children.

References	Group	Number of participants	Age (year)	Gender (male/female)
Xu et al. (20)	KD	61	2.6 ± 2.1	45/16
	Healthy control	50	2.7 ± 2.1	35/15
Wang et al. (21)	KD	19	3.2 ± 2.1	13/6
	Healthy control	56	3.2 ± 1.7	38/18
Azak et al. (22)	KD	15	4.5 ± 3.1	11/4
	Healthy control	15	5.7 ± 2.8	9/6
Wang et al. (23)	KD	50	2.1 ± 1.7	28/22
	Healthy control	30	2.2 ± 1.8	19/11
Zhang et al. (24)	KD	11	2.9 ± 1.4	7/4
	Healthy control	55	3.0 ± 1.2	35/20
Gaitonde et al. (13)	MIS-C mixed	12	8.0 ± 4.4	9/3
	KD	12	6.0 ± 2.2	8/4
Matsubara et al. (14)	MIS-C pEF	16	11.4 ± 4.2	14/14
	MIS-C dEF	12		
	KD	20	3.1 ± 1.3	15/5
	Healthy control	20	9.0 ± 2.3	11/9
Chang et al. (15)	MIS-C mixed	43	10.1 ± 4.2	26/17
	Healthy control	40	11.4 ± 3.7	23/17
Kobayashi et al. (16)	MIS-C mixed	24	11.4 ± 6.3	NR
	Healthy control	90	Age-matched	NR

*KD, Kawasaki disease; MIS-C, multisystem inflammatory syndrome in children; MIS-C dEF, Multisystem Inflammatory Syndrome in Children with depressed ejection fraction; MIS-C pEF, Multisystem Inflammatory Syndrome in Children with preserved ejection fraction; NR, nor reported.*

### Left Ventricular Ejection Fraction

In total, eight studies reported the difference of LVEF among MIS-C, KD, and healthy control groups (13–15, 20–24). MIS-C mixed group and MIS-C dEF group had a lower LVEF than KD groups (Table 2). There was no difference of LVEF among MIS-C pEF, KD, and healthy control groups.

**TABLE 2:** Head-to-head comparisons of left ventricular ejection fraction and global myocardial strain.

Left ventricular ejection fraction
**MIS-C mixed**				
−6.76 (−10.8, −2.83)	MIS-C pEF			
8.17 (3.04, 13.37)	14.94 (9.69, 20.25)	MIS-C dEF		
−10.02 (−13.2, −7.29)	−3.23 (−7.14, 0.16)	−18.2 (−23.31, −13.45)	Healthy control	
−10.00 (−13.46, −7.12)	−3.21 (−7.33, 0.23)	−18.19 (−23.47, −13.34)	0.03 (−1.73, 1.57)	KD

**Global longitudinal strain**				
**MIS-C mixed**				
2.73 (0.34, 5.11)	MIS-C pEF			
−3.17 (−5.3, −1.03)	−5.9 (−8.41, −3.37)	MIS-C dEF		
6.74 (5.38, 8.23)	4.02 (1.9, 6.27)	9.92 (8.04, 11.92)	Healthy control	
3.95 (2.47, 5.43)	1.23 (−0.99, 3.44)	7.13 (5.14, 9.09)	−2.79 (−3.73, −2.02)	KD

**Global circumferential strain**				
**MIS-C mixed**				
2.28 (−0.31, 4.79)	MIS-C pEF			
−2.28 (−5.1, 0.4)	−4.57 (−7.48, −1.71)	MIS-C dEF		
6.95 (5.16, 8.83)	4.66 (2.38, 7.15)	9.24 (6.73, 11.94)	Healthy control	
3.58 (1.56, 5.54)	1.31 (−1.16, 3.77)	5.86 (3.21, 8.57)	−3.36 (−4.68, −2.24)	KD

**Global longitudinal strain_apical four-chamber**				
**MIS-C mixed**		
3.46 (−2.33, 9.92)		Healthy control		
2.6 (−3.2, 9.67)		−0.98 (−6.55, 5.54)		KD		

**Global longitudinal strain_apical three-chamber**				
**MIS-C mixed**		
5.69 (−1.02, 12.37)		Healthy control		
2.62 (−2.84, 8.09)		−3.07 (−7.05, 0.91)		KD		

**Global longitudinal strain_apical two-chamber**				
**MIS-C mixed**		
7.67 (−2.56, 17.78)		Healthy control	
5.59 (−1.81, 12.89)		−2.07 (−9.13, 5.03)		KD	

*KD, Kawasaki disease; MIS-C, Multisystem Inflammatory Syndrome in Children; MIS-C dEF, Multisystem Inflammatory Syndrome in Children with depressed ejection fraction; MIS-C pEF, Multisystem Inflammatory Syndrome in Children with preserved ejection fraction. Data are mean difference (95% CrI) in the column-defining group compared with the row-defining group (e.g., the mean difference of left ventricular ejection fraction for MIS-mixed group compared with healthy control group is −10.02). Mean difference < 0 indicates the column-defining group had lower left ventricular ejection fraction or myocardial strain.*

### The Global Myocardial Strain

**FIGURE 3:**
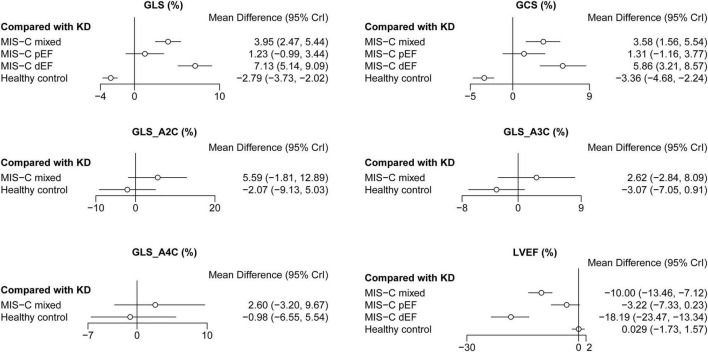
Forest plots of network meta-analysis of all studies. GCS, global circumferential strain; GLS, global longitudinal strain; GLS_A2C, global longitudinal strain_apical two-chamber; GLS_A3C, global longitudinal strain_apical three-chamber; GLS_A4C, global longitudinal strain_apical four-chamber; KD, Kawasaki disease; LVEF, left ventricular ejection fraction; MIS-C, multisystem inflammatory syndrome in children; MIS-C dEF, Multisystem Inflammatory Syndrome in Children with depressed ejection fraction; MIS-C pEF, Multisystem Inflammatory Syndrome in Children with preserved ejection fraction.

In total, eight studies reported the difference of GLS among MIS-C, KD, and healthy control groups (13–15, 20–24). Meanwhile, seven studies reported the difference of GCS among MIS-C, KD, and healthy control groups (13–15, 21–24). MIS-C mixed group and MIS-C dEF group had a higher GLS and GCS than KD groups (Figure 3). Both MIS-C pEF and KD had similar GLS and GCS, which were higher than healthy control (Table 2). There was no difference of GLS_A4C, GLS_A3C, and GLS_A2C among MIS-C mixed, KD, and healthy control groups (Figure 3).

### Inconsistency and Publication Bias

**FIGURE 4:**
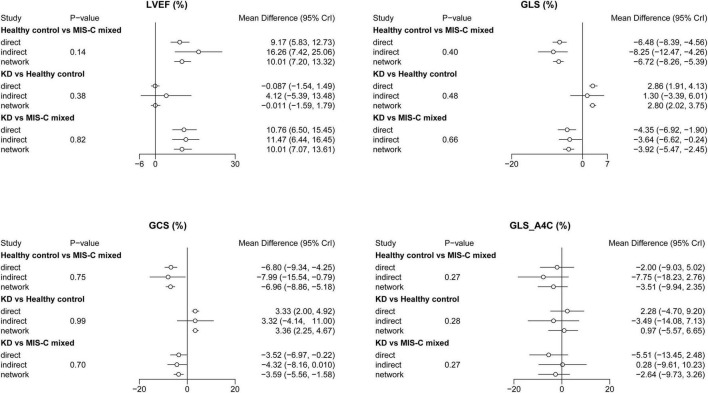
Inconsistency test of network meta-analysis. GCS, global circumferential strain; GLS, global longitudinal strain; GLS_A4C, global longitudinal strain_apical four-chamber; KD, Kawasaki disease; LVEF, left ventricular ejection fraction; MIS-C, multisystem inflammatory syndrome in children.

**FIGURE 5:**
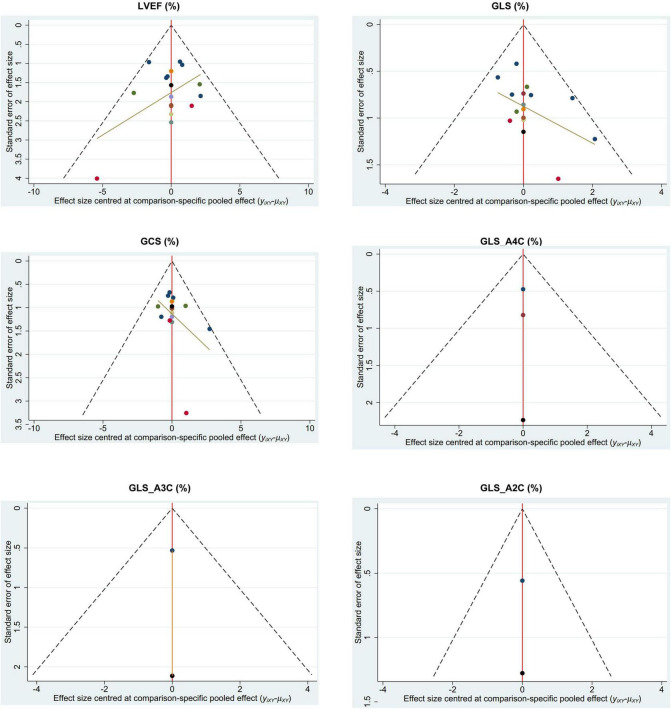
Funnel plot of network meta-analysis. GCS, global circumferential strain; GLS, global longitudinal strain; GLS_A2C, global longitudinal strain_apical two-chamber; GLS_A3C, global longitudinal strain_apical three-chamber; GLS_A4C, global longitudinal strain_apical four-chamber; LVEF, left ventricular ejection fraction.

No inconsistency result had a level of *P* < 0.05. Therefore, the results of this study were not influenced by the inconsistencies (Figure 4). The assessment of publication bias was revealed in Table 3. No obviously publication bias was detected (Figure 5).

**TABLE 3:** Assessment of publication bias.

Outcome	*P*-value of Begg’s test	*P*-value of Egger’s test
GLS	0.837	0.140
GCS	0.620	0.414
GLS_A4C	>0.999	0.312
GLS_A3C	>0.999	–
GLS_A2C	>0.999	–
LVEF	0.266	0.440

*GCS, global circumferential strain; GLS, global longitudinal strain; GLS_A2C, global longitudinal strain_apical two-chamber; GLS_A3C, global longitudinal strain_apical three-chamber; GLS_A4C, global longitudinal strain_apical four-chamber; LVEF, left ventricular ejection fraction.*

## Discussion

This is the first meta-analysis regarding the changes of myocardial strain in patients with MIS-C, compared with KD and healthy children. The results of this network meta-analysis revealed that MIS-C dEF group had lower LVEF, higher GLS and GCS than KD. MIS-C pEF group had similar LVEF, GLS, and GCS with KD. MIS-C pEF and KD had preserved LVEF, impaired GLS, and GCS in the acute phase before treatment compared with the healthy controls. In this study, GLS and GCS served as explicit indexes for MIS-C, which seems more sensitive than LVEF.

Kawasaki disease is a well-known, self-limited, systemic inflammatory vasculitis that mainly affects small- and medium-sized arteries. Several previous studies confirmed impaired left ventricular myocardial strain of KD in the acute phase using STE (20, 22) or velocity vector imaging (25, 26). Our results found that KD had impaired GLS and GCS in the acute phase compared with the healthy children, consistent with those published articles (20, 22, 25, 26). The progressive structural alterations of the myocardium and coronary arteries were confirmed during the convalescent phase of KD in the previous studies (27–29). Therefore, some studies suggested that patients with KD who had clinical recovered, need long-term follow-up to detect long-lasting impaired left ventricular myocardial strain using STE (30–33) or cardiac magnetic resonance (34).

The summarized course of myocardial strain change in patients with KD may help to better understand the recovery of MIS-C due to MIS-C having some overlapping features with KD. Before LVEF decreased or symptoms appeared, myocardial strain has emerged as a promising parameter of subclinical myocardial dysfunction (35). Myocardial strain can be measured *via* echocardiography/STE or cardiac magnetic resonance (36). However, it may be challenging to perform cardiac magnetic resonance in MIS-C because young children do not cooperate and their clinical instability (10). In order to assess the functional state of the left ventricle, STE is the most suitable technique for measuring myocardial strain (12).

Patient with MIS-C usually have fever, laboratory evidence of inflammation, and multisystem organ involvement (10). Although shares similarities with KD, MIS-C also has part features of myocarditis and toxic-shock syndrome (36). MIS-C and KD are distinct syndromes. Current evidence indicates that MIS-C is the result of an exaggerated innate and adaptive immune response, characterized by a cytokine storm, and that it is triggered by prior SARS-CoV-2 exposure (36).

The clinical course for MIS-C appears to be more severe during the acute phase. The death rate of MIS-C is 0.87% (52/5,973), compared with 0.17% of KD (4, 37). All the children who died from MIS-C had severe cardiovascular involvement (5). Recently, a study with 539 patients with MIS-C found that 65.8% of patients have preserved LVEF and 34.2% of patients have depressed LVEF (19). LVEF may become a source of heterogeneity of MIS-C studies. Therefore, as suggested by the previous research (17–19), we divided MIS-C into two subgroups. Meanwhile, we strengthened the evidence of left ventricular deformation of MIS-C by using network meta-analysis and found the difference between these two groups: left ventricular dysfunction of MIS-C dEF group was more severe than KD, but MIS-C pEF group was similar with KD in the acute phase.

Several studies focused on the recovery of MIS-C with follow-up for short-term (38, 39), mid-term (19, 40, 41), and 1-year using conventional echocardiography (42). In total, 91.0% of patients with MIS-C had a normal LVEF by 30 days (19). LVEF of all patients with MIS-C needed 74–90 days to return to normal (19, 42). However, cardiac magnetic resonance studies did not find any abnormality in patients with MIS-C after 27 ± 14 days (43), or a 2-month follow-up (44). In our involved study, three studies performed the short-term follow-up (13, 14, 16). After treatment, LVEF and GCS normalized by 7–9 days of illness, impaired GLS persisted in most of the patients with MIS-C (14, 16), similar findings were reported by a recent MIS-C cohort study (45). GLS may be a good follow-up index for MIS-C. Thus, we recommend long-term, regular intervals, follow-up with myocardial strain echocardiography for MIS-C, supplemented with cardiac magnetic resonance if clinical conditions permit.

Global longitudinal strain is usually associated with adverse outcomes, especially in patients with myocarditis (46), heart failure (47), or cardiomyopathy (48). GLS may be a clinical predictor for MIS-C. In patients with higher GLS, they have higher risk of intensive care, mechanical ventilation, or inotropic support (16, 18, 45). Meanwhile, they may stay longer in intensive care unit or hospital (18, 45). Meanwhile, correlations of GLS with laboratory parameters in patients with MIS-C were reported, such as C-reactive protein, troponin, and brain natriuretic peptide, indicating the presence of myocarditis and cardiac dysfunction (13, 16, 45, 49). Therefore, the impaired myocardial strain on the initial echocardiography of patients presenting with KD-like illness may be a clue to differential diagnosis. Such patients require closer clinical observation for the development of cardiac dysfunction (13).

### Limitation

First, this analysis was performed based on observational studies. The small sample size cannot be ignored. Not all involved studies mentioned this index of myocardial strain were normal distribution. All the aforementioned will affect the reliability of the results. Second, some underlying confounders, such as age, gender, sex, and body mass index, may become that were not adjustable. Third, no mid-term or long-term follow-up in all involved studies. Fourth, none of the involved studies focused on the potential impact of the coronary aneurysm on MIS-C. Fifth, the myocardial strain values may be measured by different echocardiography machines from the different manufacturers.

## Conclusion

MIS-C dEF was more severe than KD, both in LVEF and global myocardial strain. MIS-C pEF and KD were similar with mild-impaired left ventricular myocardial strain, compared with the healthy children. Global myocardial strain may be a monitoring index for MIS-C.

## Data Availability Statement

The original contributions presented in this study are included in the article/Supplementary Material, further inquiries can be directed to the corresponding author.

## Author Contributions

KL contributed to the data collection, data analysis, and manuscript writing. JY contributed to the data collection and data analysis. GS contributed to the project development and manuscript writing. All authors contributed intellectually to the work and read and approved the final manuscript.

## Conflict of Interest

The authors declare that the research was conducted in the absence of any commercial or financial relationships that could be construed as a potential conflict of interest.

## Publisher’s Note

All claims expressed in this article are solely those of the authors and do not necessarily represent those of their affiliated organizations, or those of the publisher, the editors and the reviewers. Any product that may be evaluated in this article, or claim that may be made by its manufacturer, is not guaranteed or endorsed by the publisher.
